# Supplementary material for: Two-Step Preparation of CCF/PEEK Wrapped Yarn for 3D Printing Composites with Enhanced Mechanical Properties
Source: Materials (Basel). 2023 Jan 30;16(3):1168. doi: 10.3390/ma16031168 (PMC9921352; doi:10.3390/ma16031168)
Supplement: Supplementary file 1 [file materials-16-01168-s001.zip › materials-2180674-supplementary.pdf]

Supporting information

Two-Step Preparation of CCF/PEEK Wrapped Yarn for 3D Printing Composites with Enhanced Mechanical Properties

Jianghu Zhang [1,2], Hao Shen [2], Lili Yang [1,*], Dengteng Ge [2,*]

[1] State Key Laboratory for Modification of Chemical Fibers and Polymer Materials, College of Materials Science and Engineering, Donghua University, Shanghai 201620, China

[2] Institute of Functional Materials, Donghua University, Shanghai 201620, China

This PDF includes:

Figure S1 to Figure S4

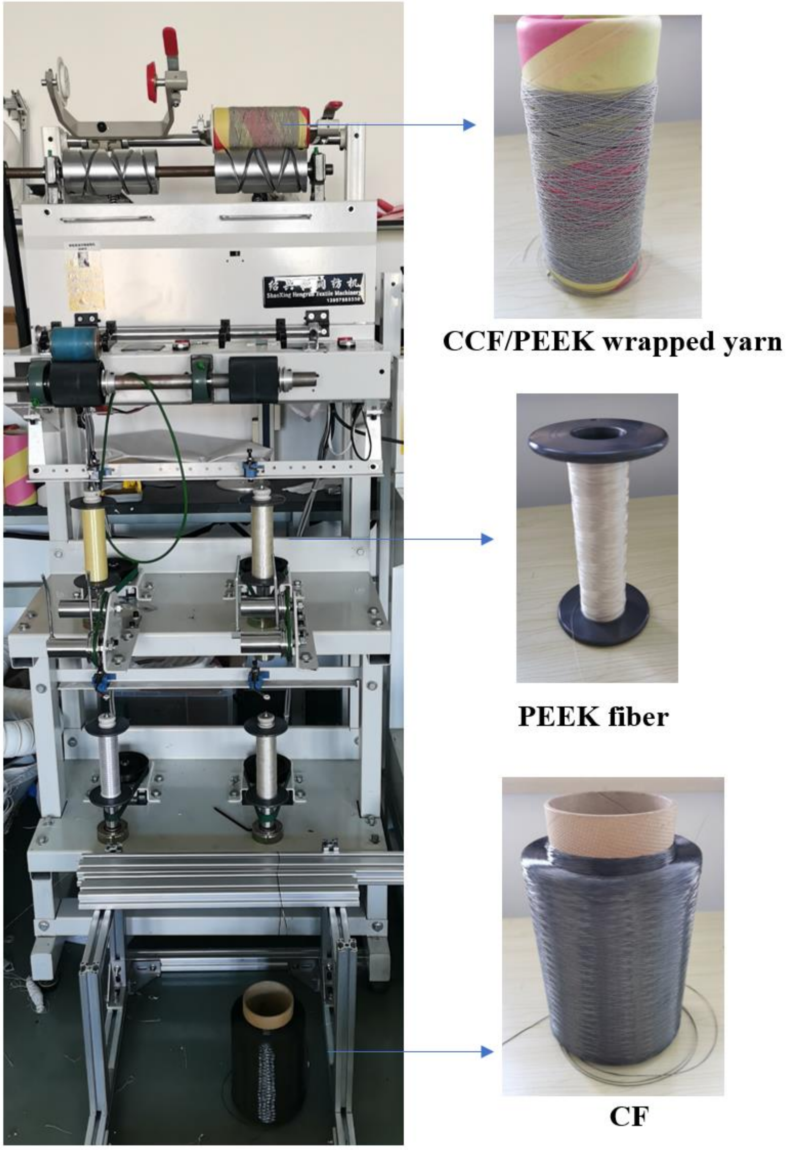

Figure S1. Pictures of equipment for the double spinning process of wrapped yarn.

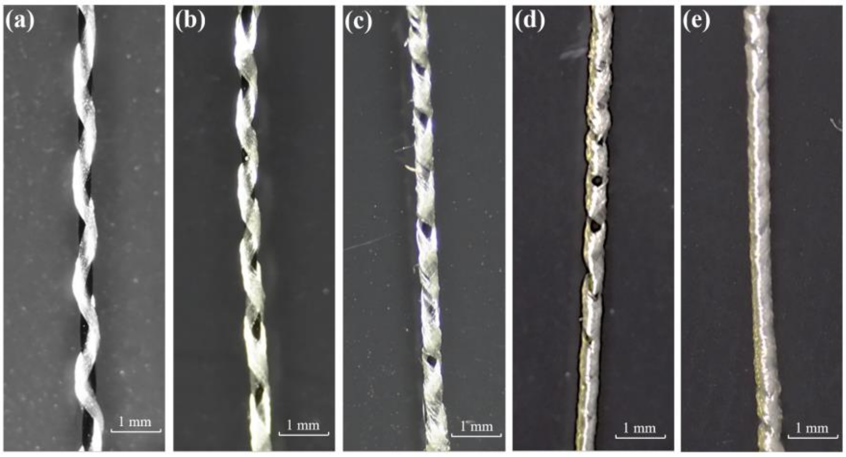

Figure S2. Photos of CCF/PEEK wrapped yarn at different winding rates of (**a**) 2800 r/min, (**b**) 4200 r/min, (**c**) 5600 r/min, (**d**) 7000 r/min, (**e**) 8400 r/min and constant CF wire speed of 6.75 m/min.

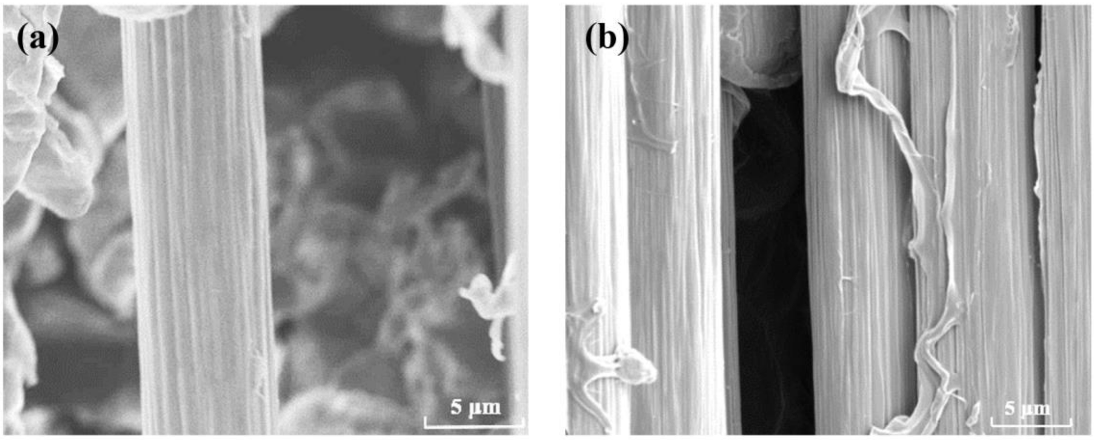

Figure S3. High resolution SEM images of (**a**) powder impregnated CCF/PEEK wrapped yarn and that (**b**) after desizing.

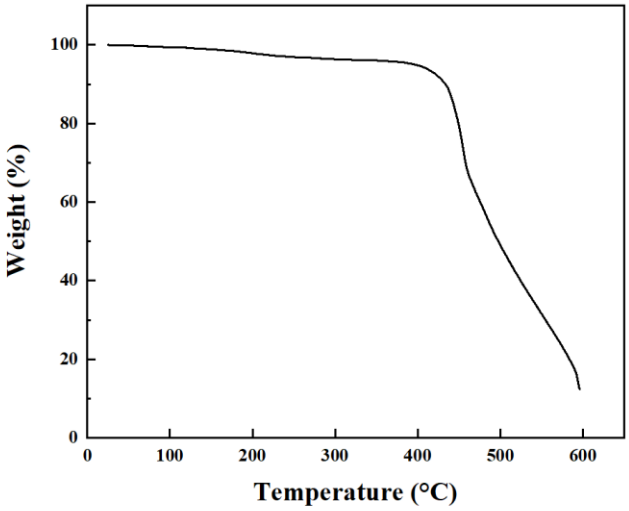

Figure S4. TG curve of PEEK fiber.
